# Association between intake of less-healthy foods defined by the United Kingdom's nutrient profile model and cardiovascular disease: A population-based cohort study

**DOI:** 10.1371/journal.pmed.1002484

**Published:** 2018-01-04

**Authors:** Oliver T. Mytton, Nita G. Forouhi, Peter Scarborough, Marleen Lentjes, Robert Luben, Mike Rayner, Kay Tee Khaw, Nicholas J. Wareham, Pablo Monsivais

**Affiliations:** 1 UKCRC Centre for Diet and Activity Research, MRC Epidemiology Unit, University of Cambridge, Cambridge, United Kingdom; 2 Centre on Population Approaches for Non-Communicable Disease Prevention, Nuffield Department of Population Health, University of Oxford, Oxford, United Kingdom; 3 Strangeways Research Laboratories, Department of Public Health and Primary Care, University of Cambridge, Cambridge, United Kingdom; 4 Department of Nutrition and Exercise Physiology, Washington State University, Spokane, Washington, United States of America; Carolina Population Center, UNITED STATES

## Abstract

**Background:**

In the United Kingdom, the Food Standards Agency-Ofcom nutrient profiling model (FSA-Ofcom model) is used to define less-healthy foods that cannot be advertised to children. However, there has been limited investigation of whether less-healthy foods defined by this model are associated with prospective health outcomes. The objective of this study was to test whether consumption of less-healthy food as defined by the FSA-Ofcom model is associated with cardiovascular disease (CVD).

**Methods and findings:**

We used data from the European Prospective Investigation of Cancer (EPIC)-Norfolk cohort study in adults (*n* = 25,639) aged 40–79 years who completed a 7-day diet diary between 1993 and 1997. Incident CVD (primary outcome), cardiovascular mortality, and all-cause mortality (secondary outcomes) were identified using record linkage to hospital admissions data and death certificates up to 31 March 2015. Each food and beverage item reported was coded and given a continuous score, using the FSA-Ofcom model, based on the consumption of energy; saturated fat; total sugar; sodium; nonsoluble fibre; protein; and fruits, vegetables, and nuts. Items were classified as less-healthy using Ofcom regulation thresholds. We used Cox proportional hazards regression to test for an association between consumption of less-healthy food and incident CVD. Sensitivity analyses explored whether the results differed based on the definition of the exposure. Analyses were adjusted for age, sex, behavioural risk factors, clinical risk factors, and socioeconomic status. Participants were followed up for a mean of 16.4 years. During follow-up, there were 4,965 incident cases of CVD (1,524 fatal within 30 days). In the unadjusted analyses, we observed an association between consumption of less-healthy food and incident CVD (test for linear trend over quintile groups, *p* < 0.01). After adjustment for covariates (sociodemographic, behavioural, and indices of cardiovascular risk), we found no association between consumption of less-healthy food and incident CVD (*p* = 0.84) or cardiovascular mortality (*p* = 0.90), but there was an association between consumption of less-healthy food and all-cause mortality (test for linear trend, *p* = 0.006; quintile group 5, highest consumption of less-healthy food, versus quintile group 1, HR = 1.11, 95% CI 1.02–1.20). Sensitivity analyses produced similar results. The study is observational and relies on self-report of dietary consumption. Despite adjustment for known and reported confounders, residual confounding is possible.

**Conclusions:**

After adjustment for potential confounding factors, no significant association between consumption of less-healthy food (as classified by the FSA-Ofcom model) and CVD was observed in this study. This suggests, in the UK setting, that the FSA-Ofcom model is not consistently discriminating among foods with respect to their association with CVD. More studies are needed to understand better the relationship between consumption of less-healthy food, defined by the FSA-Ofcom model, and indices of health.

## Abstract 2

Using data from the EPIC-Norfolk cohort study, Oliver Mytton and colleagues examine the association between intake of less healthy foods defined by the UK's nutrient profile model and cardiovascular disease.

## Author summary

**Why was this study done?:**

- The Food Standards Agency (FSA)-Ofcom model is used in the UK to identify ‘less-healthy’ foods in order to restrict their advertising to children.
- Variants of the FSA-Ofcom model, as well as other nutrient profiling models, are increasingly being used to regulate food retailing or marketing for the purposes of improving health; yet, very few of these models have been validated.
- The FSA-Ofcom model has been shown to classify foods in a way that is consistent with professional opinion, but there has been limited assessment of its association with health outcomes.

**What did the researchers do and find?:**

- We used the European Prospective Investigation of Cancer (EPIC)-Norfolk study to test the prospective association of less-healthy food consumption with incident cardiovascular disease, cardiovascular mortality, and all-cause mortality.
- Each item of food or drink reported in a participant’s 7-day diet diary was given a score based on its nutrient composition and then categorised as either ‘less-healthy’ or ‘healthy’.
- Participants (*n* = 22,292) were allocated to 1 of 5 groups based on their consumption of less-healthy food (as a proportion of total dietary energy).
- After adjustment for confounding factors, we found no association between consumption of less-healthy food and incident cardiovascular disease (*n* = 4,965) or cardiovascular mortality (*n* = 2,555)
- The findings were robust to a variety of sensitivity analyses, including adjustment for exclusion based on different cardiovascular risk factors.

**What do these findings mean?:**

- Whilst no single study is definitive and our findings are in contrast to similar work in a French cohort, these findings suggest that the FSA-Ofcom model is not consistently discriminating among foods with respect to their associations with cardiovascular disease in the UK context.
- Public health officials and scientists may want to review whether and how the FSA-Ofcom scoring system could be improved for use in the UK and elsewhere.
- There is a robust evidence base concerning the health risks associated with the consumption of many foods that are often labelled ‘unhealthy’ (e.g., red meat, sugar-sweetened beverages, and takeaway food), and it would be inappropriate to use this study to undermine present dietary advice for the public.

## Introduction

Nutrient profiling is the science of classifying or ranking foods according to their nutritional composition for reasons related to preventing disease [1,2]. Over 100 nutrient profile models exist globally (around 60 of which are publicly available). One of the most prominent is a model originally devised by the Food Standards Agency (FSA) and used in the UK by the communications regulator (Ofcom) to restrict the advertising of unhealthy foods to children [3–5]. Variations on this model have been used in other countries (e.g., in Australia, New Zealand, France, and South Africa) [1,6,7].

The FSA-Ofcom model has 2 parts: a scoring system that assigns each food item a numerical score based on its nutrient composition and a classification system that then categorises each food or beverage item that exceeds a prespecified score as ‘less-healthy’. Ranking foods by the FSA-Ofcom model has been shown to correlate with the views of nutritional professionals, and classifications compare favourably with UK food-based dietary guidelines [8,9]. In two French cohorts, prospective associations between a diet consisting of foods with a higher mean score and weight gain, development of metabolic syndrome, cardiovascular risk, and cancer risk have been reported [10–14]. There are no similar studies in a UK population. The French studies do not reflect how the model is used in the UK presently. The French scoring system is similar to that of the FSA-Ofcom model but scores fats, cheeses, and beverages differently [13,15], and the French studies have tested the scoring system rather than the classification system. There may also be important differences between French and British diets [16,17], which could result in different associations.

Our objective was to test whether consumption of less-healthy food, as identified by the FSA-Ofcom model, was associated with incident cardiovascular disease (ischaemic heart disease and stroke). We chose to focus on cardiovascular disease (CVD) because the components of the scoring system (e.g., saturated fat, salt, sugar, fruits, and vegetables) suggest that it should identify foods that would be associated with a higher risk of CVD.

## Methods

### Ethics statement

The EPIC-Norfolk study protocol was approved by the Norwich District Health Authority Ethics Committee, and all participants gave written informed consent.

### Study population

The EPIC-Norfolk study is part of the European Prospective Investigation of Cancer (EPIC) study that spans 10 European countries. It has been described in detail elsewhere [18]. In brief, participants aged 40–79 years were recruited from the general population through general practices in the east of England between 1993 and 1997. Participants (*n* = 25,639) completed a baseline questionnaire covering sociodemographic factors, medical history, medication use and health behaviours, completed a 7-day diet diary [19], and attended a clinical research facility (for measurement of blood pressure, height, and weight). Health outcomes were ascertained by linkage to hospital admissions data and death certificates.

### Exclusion criteria

We excluded participants who did not complete at least 1 day of the 7-day diet diary and those who were in the top or bottom 0.5% of the distribution of the ratio of reported energy intake to basal metabolic rate (calculated using sex-specific Schofield equations) [20]. For analysis of incident CVD, we further excluded participants with prevalent disease (self-reported angina, heart attack, or stroke) as well as those with missing covariates. For analysis of mortality, we included participants with prevalent disease and excluded participants with missing covariates. Because missing covariate data were limited to a small proportion of the total sample (1.08%, 250/23,242, for analysis of incident CVD; 1.29%, 322/24,880, for analysis of mortality outcomes), we chose to exclude these participants rather than impute missing data.

### Dietary assessment

Participants reported their food intake for 1 week using a 7-day diet diary. A trained nurse, during the visit to the clinical research facility, obtained a 24-hour-diet recall that formed the first day of the diet diary and served as a general instruction regarding the detail required for the diary. Participants were additionally provided with written instructions, and the diet diary contained colour photographs to aid portion size estimation [19,21]. The 7-day diet diaries were entered using the in-house developed DINER data-entry system and checked and calculated using the DINERMO processing programmes [22,23]. For each food item, we also ascertained the proportion (by weight) that was fruit, vegetables, pulses/lentils, or nuts, which we have previously described as ‘disaggregated food groups’ [23]. This resulted in nutrient quantities and (disaggregated) food weight intake for every food item consumed. The majority of included participants (90.8%; 20,885/22,992) completed all 7 days of the diary.

### Nutrient profile score

The FSA-Ofcom model assigns an overall numeric score for any given item of food, based on the following components: energy; saturated fat; total sugar; sodium; nonsoluble fibre; protein; and fruit, vegetable, and nut content. In summary, each component is scored based on the quantity per 100 g edible weight [24]. Scores for energy, saturated fat, total sugar, and sodium are positive (i.e., adverse score), graded on a 10-point scale. Scores for nonsoluble fibre and protein as well as fruits, vegetables, and nuts are negative (i.e., beneficial or healthy score), graded on a 5-point scale. A copy of the full algorithm is available for download [24] and outlines how the scores for the different components are added together to give the overall score. If a food scores 4 points or more, it is categorised as less-healthy, and a beverage is categorised as less-healthy if it scores 1 point or more. Reflecting the operational use of the FSA-Ofcom model, any beverage that contained alcohol was not scored [10,25,26].

### Classification of exposure

For each participant, we summed the energy consumed from all foods and beverages (referred to as ‘food items’) that were classified as less-healthy. Energy from alcoholic beverages formed a separate group, since alcohol is not part of the score guidelines. For each participant, we estimated the proportion of energy consumed from food items that were classified as less-healthy by the FSA-Ofcom model:

$$\frac{\left(Energy\;from\;less\text{‑}healthy\;food+Energy\;from\;less\text{‑}healthy\;beverages\right)}{\left(Total\;energy\;intake-Energy\;alcoholic\;beverages\right)}$$

We then divided the study sample into quintile groups (fifths) based on this proportion. Thus, our primary exposure measure was quintile groups of proportion of energy intake consumed from food items categorised as less healthy.

### Outcome ascertainment

Our primary outcome measure was incident CVD. Secondary outcome measures were cardiovascular mortality and total (all-cause) mortality.

We defined incident cases of CVD as any primary fatal or nonfatal event of ischaemic heart disease (International Classification of Disease [ICD]-10 codes I20–I25) or cerebrovascular disease (stroke) (ICD-10 codes I60–I69). Incident cases were ascertained by record linkage to hospital admissions data and death certificates coded for CVD using the ICD-10 criteria. Death from any cause, including cardiovascular death, was ascertained by record linkage to mortality data confirmed via death certificates with ICD codes held at the UK Office for National Statistics. Record linkage for deaths and hospital admissions was complete to 31 March 2015.

### Statistical analysis

We used Cox proportional hazards regression to estimate the hazard ratio and 95% confidence interval for the association between exposure and outcome. Whilst aspects of the analytic plan (e.g., classification of exposure, choice of outcomes, and use of Cox proportional hazards) were agreed prior to beginning the analysis (S1 Text), there was no preagreed study protocol specifying the choice of covariates and sensitivity analyses.

We adjusted analyses for two sets of potential confounders. Information on other covariates was obtained from the baseline questionnaire. Model 1 was adjusted for sociodemographic and behavioural risk factors: age (continuous, years), sex, level of education, smoking status (never, former, or current), physical activity (inactive, moderately inactive, moderately active, or active), alcohol consumption (units/day), and overall energy intake (kJ/day). Model 2 additionally adjusted for self-reported clinical risk factors at baseline (blood pressure-lowering medication, lipid-lowering medication, prevalent diabetes, prevalent hypertension, prevalent hypercholesterolemia, past cancer diagnosis, family history of myocardial infarction, family history of stroke, and family history of diabetes).

The decision to include an extensive list of possible confounders in a second model was made after the descriptive analyses showed evidence of increased cardiovascular risk amongst participants who were consuming the least amount of less-healthy food (i.e., possible reverse causation) and because of the failure of the original analytic analyses to demonstrate an association between increasing consumption of less-healthy food and CVD (which might be attributable to reverse causation). We adjusted for indicators that were likely to signal cardiovascular risk to the participant (rather than all measures of cardiovascular risk), as these might influence dietary behaviour (e.g., knowing that one has a diagnosis of hypertension might affect dietary behaviour). In practice, this meant adjusting for self-reported diagnoses (hypertension, hyperlipidaemia, diabetes, and cancer), reported medication usage (for blood pressure and cholesterol), and reported family history (ischaemic heart disease, stroke, and diabetes). These factors are causally related to incident CVD and, given the descriptive data, might contribute to reverse causation. We did not adjust for factors that might be unknown by the participant and might be on the causal pathway between diet and disease (e.g., measured blood pressure and measured cholesterol). While some of the covariates included in Model 2 may act as confounders, they may also be on the causal pathway, i.e., act as mediators (e.g., poor diet leading to hypertension leading to CVD), and thus, adjustment for these factors might be considered overadjustment. In response to comments from peer review, we additionally report Model 2’, which excludes potential mediators, i.e., adjusts for Model 1 covariates, past cancer diagnosis, family history of myocardial infarction, family history of stroke, and family history of diabetes.

In analyses assessing the outcome of mortality, we additionally adjusted for prevalent CVD (self-reported angina, stroke, and heart attack).

To aid interpretation and as a test of an increasing trend across quintiles, we report the significance of the regression coefficient for the quintiled exposure when it was treated as a continuous variable. All analyses were conducted in Stata v13. We used visual plots and Schoenfeld residuals to test the proportional hazards assumption.

In addition, we also tested the association between quintile group of fruit and vegetable consumption (ranked on weight consumed), adjusting for the same set of covariates. Associations between fruit and vegetable consumption and CVD [27–29] are commonly observed, so an association would be expected. This analysis served as a validation of the approach to categorisation of the exposure and the analytic approach. The decision to include this analysis was made retrospectively in light of the initial findings.

### Sensitivity analyses

We undertook the following sensitivity analyses. First, in light of initial findings, we repeated our primary analysis of combined CVD as an outcome with the separate outcomes of incident myocardial infarction and incident stroke. Second, in response to comments from peer review, we repeated the analysis but did not adjust for total dietary intake. This is sometimes considered appropriate when testing the relationship between dietary patterns and disease if it is thought that dietary patterns mediate their effect on disease through total energy intake.

Third, we used different approaches to the categorisation of less-healthy food consumption: (A) We allocated participants to a quintile group based on the proportion of food weight that was categorised as less-healthy (rather than food and beverage energy, since the relatively high weight of beverages might distort any association; this analysis was preplanned), and (B) we allocated participants to a quintile group based on the mean energy-weighted FSA-Ofcom score of all food items consumed. This latter approach is the same as that used by other authors and was introduced in response to work published after the study was conceived [10–13]. It effectively only tested the first part of the FSA-Ofcom model, the scoring system, treating it as a ‘dietary index’ measure, and did not test the classification system. In addition and in response to comments from peer review, we tested a ‘substitution model’ in which we included the following terms: energy from unhealthy food, energy from unhealthy beverages, energy from healthy beverages, and total dietary energy. The resultant coefficient estimates the hazard ratio when energy from unhealthy food is replaced with energy from healthy food, holding total energy intake constant.

Fourth, we took an alternative approach to confounding variables: (A) After undertaking the initial analysis and noting the inverse association between body mass index (BMI) and consumption of less-healthy food, we additionally adjusted the primary analysis for baseline BMI; and (B) to test for residual confounding by prevalent disease within the mortality analyses, we repeated the mortality analyses excluding participants with prevalent CVD (self-reported angina, stroke, and heart attack). In response to comments from peer review, we have introduced a further set of analyses to address potential reverse causation. First, we excluded all events that occurred within 2 years of follow-up. Second, we excluded—rather than adjusted for—comorbidities at baseline, excluding participants with cardiovascular comorbidities (self-reported hypertension, hyperlipidaemia, blood pressure medication, or lipid-lowering medication) or those with other comorbidities (diabetes and cancer). Third, we excluded participants with a family history of CVD (stroke or heart attack). Finally, we combined all these exclusion criteria and additionally excluded participants with a family history of diabetes, thus restricting the analysis to participants with no reported comorbidities at baseline, with no reported family history of CVD or diabetes, and who did not have an incident event within 2 years of follow-up.

## Results

**Fig 1:**
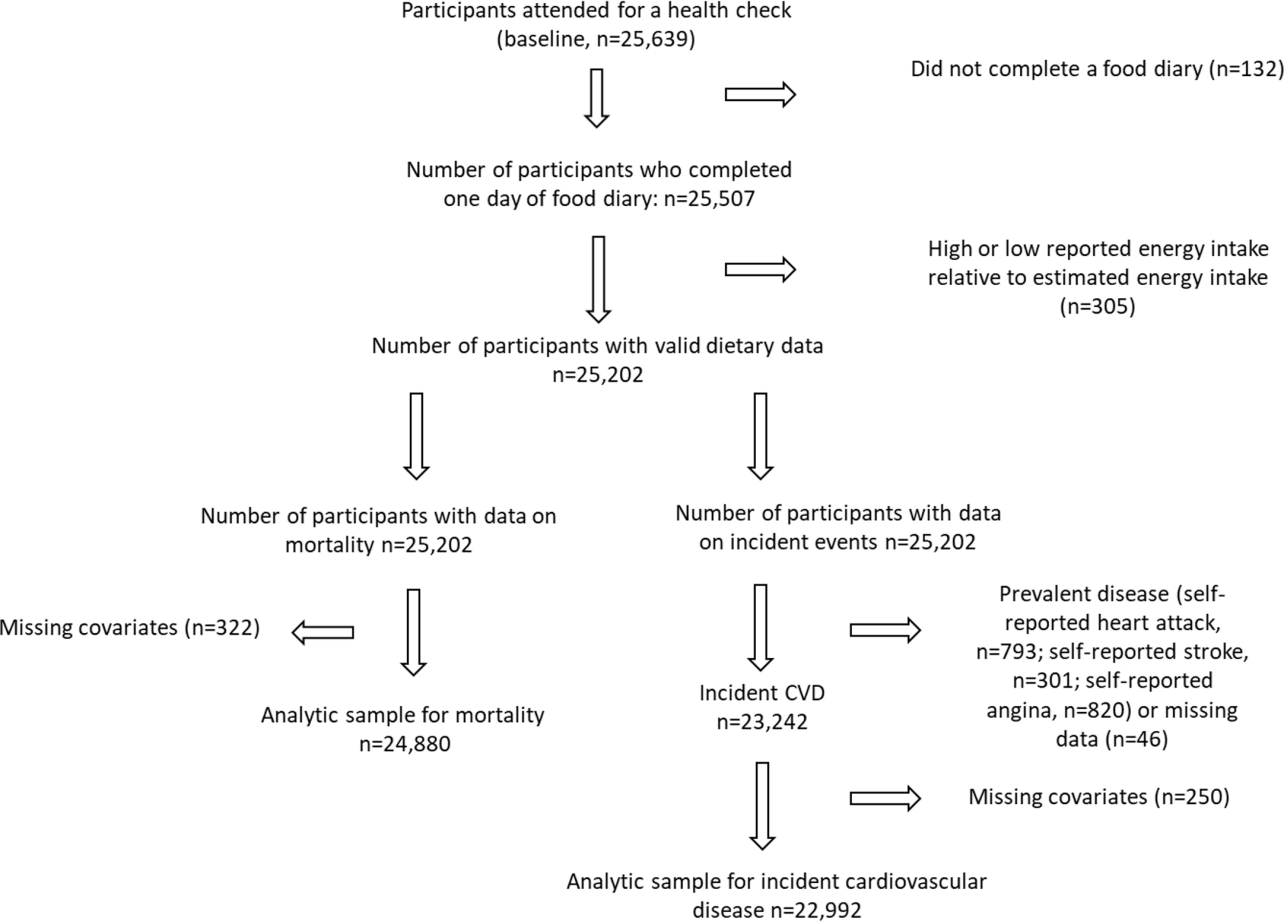
Flow diagram summarising participants included in the analysis.

After exclusions (Fig 1), there were 22,992 participants included in the analyses of incident CVD and 24,880 in the analyses of mortality. There were no important differences in the baseline characteristics of participants included and excluded because of missing covariates (Table A in S1 Data). Participants were followed up for a mean of 16.4 years. During follow-up, there were 4,965 incident cases of CVD (1,524 fatal within 30 days). Among a total of 7,139 all-cause deaths, 2,555 deaths were attributed to CVD. The baseline characteristics of the participants are shown in Table 1. Those in quintile group 5 (i.e., highest proportional consumption of less-healthy food) were more likely to be older and male and less likely to have completed higher education (degree or equivalent). Some health indices among quintile group 5 were worse—for example, a greater proportion of participants reported being current smokers. However, some health indices were better—for example, they were less likely to be on medication (antihypertensives or lipid-lowering medication), were less likely to have a family history of heart attack, and had a lower BMI. Reported physical activity did not differ appreciably across the quintile groups.

**Table 1:** Baseline characteristics of participants: The European Prospective Investigation of Cancer (EPIC)-Norfolk study (*n* = 22,992).

	Quintile group of less-healthy food and beverage consumption (proportion of energy consumed from foods and beverages categorised as ‘less-healthy’)					Total sample (*n* = 22,992)
	Q1—lowest (*n* = 4,599)	Q2 (*n* = 4,598)	Q3 (*n* = 4,599)	Q4 (*n* = 4,598)	Q5—highest (*n* = 4,598)	
**Sociodemographic, behavioural, and medical risk factors**						
Age (years)	57.7 (8.8)	58.2 (8.9)	58.9 (9.2)	59.1 (9.4)	59.2 (9.6)	58.6 (9.2)
Women (%)	63.5	58.7	57.8	53.7	48.5	56.5
Education: degree or higher (%)	16.0	13.9	13.6	12.5	10.9	13.4
Current smoker (%)	10.4	11.2	10.5	11.0	15.8	11.8
Physical activity: active (%)	18.7	18.1	18.4	19.1	19.3	18.7
Past cancer diagnosis (%)	5.3	5.4	5.3	5.5	5.6	5.4
Diabetes (%)	3.3	2.2	1.7	1.2	1.0	1.9
Family history of heart attack (%)	37.5	36.0	34.6	35.7	34.0	35.6
Antihypertensive medication (%)	16.2	15.8	14.6	14.0	13.9	14.9
Lipid-lowering medication (%)	1.4	1.1	0.8	0.7	0.2	0.9
BMI (kg/m^2^)	26.7 (4.1)	26.4 (3.9)	26.2 (3.8)	26.2 (3.8)	25.9 (3.80)	26.3 (3.9)
Systolic blood pressure (mmHg)	135.2 (18.9)	134.9 (18.0)	135.5 (18.5)	135.0 (18.0)	135.3 (18.4)	135.2 (18.4)
Total cholesterol (mmol/L)	6.13 (1.18)	6.18 (1.15)	6.18 (1.14)	6.19 (1.16)	6.14 (1.17)	6.17 (1.16)
HDL cholesterol (mmol/L)	1.47 (0.42)	1.45 (0.47)	1.44 (0.42)	1.41 (0.41)	1.38 (0.40)	1.43 (0.42)
**Measures of dietary quality (mean consumption per day)**						
Fruit (g)	218 (169)	187 (135)	171 (125)	156 (113)	132 (106)	173 (134)
Vegetables (g)	178 (95)	160 (74)	151 (71)	141 (66)	127 (68)	152 (77)
Fish (g)	32.5 (33.0)	29.7 (27.2)	27.8 (26.4)	25.1 (23.8)	22.0 (24.6)	27.4 (27.4)
Processed meat (g)	17.4 (18.9)	21.3 (19.8)	22.5 (19.9)	24.3 (21.2)	25.9 (24.1)	22.3 (21.0)
Alcohol (units)	2.35 (3.12)	1.86 (2.35)	1.48 (1.91)	1.13 (1.56)	0.72 (1.15)	1.51 (2.21)
Energy (kJ)	7,210 (2,020)	7,895 (2,025)	8,242 (2,016)	8,606 (2,079)	9,121 (2,309)	8217 (2192)
Percentage of energy from saturated fat (%)	10.2 (2.5)	12.1 (2.4)	13.0 (2.4)	13.8 (2.6)	15.2 (3.0)	12.9 (3.1)
Ratio of saturated to unsaturated fat	1.81 (0.72)	1.99 (0.69)	2.11 (0.77)	2.23 (0.82)	2.52 (1.01)	2.13 (0.84)
Sodium (mg)	2,400 (780)	2,690 (820)	2,770 (790)	2,900 (810)	3,020 (900)	2,760 (850)
Fibre (g)	16.2 (6.3)	15.5 (5.4)	15.1 (5.4)	14.7 (5.1)	13.9 (5.2)	15.1 (5.5)
**Characteristics of diet defined by FSA-Ofcom model**						
Mean energy-weighted nutrient profile score	3.99 (1.52)	5.99 (1.10)	7.05 (1.12)	8.07 (1.21)	9.50 (1.56)	6.91 (2.28)
Less-healthy food (g/d)	181 (76)	258 (84)	298 (87.5)	343 (99)	405.5 (125)	297.3 (122)
Less-healthy food (kJ/d)	2,091 (795)	3,109 (850)	3,716 (959)	4,355 (1,133)	5,319 (1,498)	3,718 (1,535)
Healthy food (kJ/d)	3,973 (1,113)	3,717 (965)	3,508 (893)	3,312 (838)	2,891 (870)	3480 (1010)
Less-healthy beverage (kJ/d)	81 (153)	133 (213)	177.5 (265)	246.2 (338)	435 (501)	216 (342)

Values shown are the percentage for categorical data and the mean (standard deviation) for continuous data. Physical activity = percentage who are classified as ’active’, i.e., meeting guidelines for recommended amount of moderate-to-vigorous physical activity. Medical history (cancer diagnosis, diabetes, and medication) was self-reported. The estimates of dietary intake are derived from the 7-day diet diary. The mean nutrient profile score is the mean-energy-weighted nutrient profile score of all foods and nonalcoholic beverage measured using the FSA-Ofcom scoring system. Less-healthy food is food with a nutrient profile score of 4 points or more; a less-healthy beverage scores 1 point or more. BMI, body mass index; FSA, Food Standards Agency.

The quality of diet as assessed by different foods and nutrients showed a gradient across the quintile groups, with those who consumed the highest proportion of less-healthy food also consuming higher absolute quantities of foods or nutrients associated with poor health (e.g., salt, processed meat, saturated fat, and sodium) and lower absolute quantities of foods or nutrients associated with good health (e.g., fish, fruit, and vegetables, as well as a lower ratio of polyunsaturated to saturated fat) (see Table 1). Individuals in quintile group 5 also consumed more energy. At baseline, those in quintile group 5 consumed over twice as much less-healthy food and over 5 times as many less-healthy beverages in comparison to those in quintile group 1.

### Prospective associations with health end points

Table 2 shows the prospective associations between quintile groups of proportional less-healthy food consumption and incident CVD. The unadjusted analyses showed a positive association between consumption of less-healthy food and incident CVD. After adjustment for sociodemographic and behavioural factors (Model 1), there was an inverse (protective association) (test for trend, *p* = 0.009) between consumption of less-healthy food and incident CVD. After additional adjustment for indicators of cardiovascular risk at baseline (Model 2), there was no association between less-healthy food consumption and incident CVD. The same pattern of findings was observed when we took a different approach to adjustment for confounders, additionally adjusting for BMI (Model 2 + BMI, Table B in S1 Data) or adjusting for a more restricted set of indices of cardiovascular risk, (Model 2′, Table B in S1 Data).

**Table 2:** Cox regression models for incident cardiovascular disease in the European Prospective Investigation of Cancer (EPIC)-Norfolk (*n* = 22,992).

		Hazard ratio (95% CI)		
		Unadjusted	Model 1	Model 2
Quintile group of proportional energy provided by less-healthy food consumption	Q1 (reference; lowest)	1.00	1.00	1.00
	Q2	1.04 (0.95–1.14)	0.97 (0.88–1.06)	0.99 (0.91–1.08)
	Q3	1.07 (0.98–1.17)	0.93 (0.85–1.02)	0.99 (0.90–1.08)
	Q4	1.09 (0.99–1.19)	**0.90 (0.82–0.99)**	0.97 (0.88–1.07)
	Q5 (highest)	**1.19 (1.10–1.31)**	0.93 (0.84–1.03)	1.01 (0.92–1.12)
	Test for linear trend (*p*-value)	**0.007**	**0.009**	0.84
Age (per year)			**1.09 (1.09–1.09)**	**1.08 (1.08–1.09)**
Sex (reference = male)			**0.53 (0.49–0.56)**	**0.52 (0.48–0.55)**
Alcohol (per unit/d)			0.98 (0.97–1.00)	0.99 (0.97–1.00)
Physical activity	Inactive (reference)		1.00	1.00
	Moderately inactive		**0.84 (0.79–0.91)**	**0.86 (0.80–0.92)**
	Moderately active		**0.84 (0.77–0.91)**	**0.87 (0.80–0.94)**
	Active		**0.83 (0.76–0.91)**	**0.86 (0.79–0.94)**
Cigarette smoking	Current (reference)		1.00	1.00
	Exsmoker		**0.66 (0.60–0.72)**	**0.63 (0.58–0.69)**
	Never smoker		**0.58 (0.53–0.63)**	**0.56 (0.51–0.61)**
Highest education qualification	No qualifications (reference)		1.00	1.00
	O-Level or equivalent		**0.83 (0.75–0.93)**	**0.83 (0.75–0.93)**
	A-Level or equivalent		**0.87 (0.82–0.93)**	**0.88 (0.82–0.93)**
	Degree or equivalent		**0.76 (0.69–0.84)**	**0.77 (0.69–0.85)**
Energy (per 2,000 kJ/d)			**0.95 (0.91–0.98)**	**0.95 (0.92–0.99)**
Antihypertensive medication				**1.35 (1.23–1.48)**
Lipid-lowering medication				0.78 (0.59–1.03)
Past cancer diagnosis				**1.19 (1.06–1.34)**
Diabetes				**1.89 (1.64–2.18)**
Hypertension				**1.23 (1.12–1.35)**
Hypercholesterolemia				**1.36 (1.23–1.50)**
Family history of heart attack				**1.19 (1.13–1.26)**
Family history of stroke				**1.07 (1.01–1.14)**
Family history of diabetes				1.09 (1.00–1.19)

Model 1 is adjusted for age, sex, alcohol consumption, physical activity, smoking status, education level, and total dietary energy. Model 2 is adjusted for Model 1 covariates plus blood pressure-lowering medication, lipid-lowering medication, diabetes, hypertension, hypercholesterolemia, past cancer diagnosis, family history of heart attack, family history of stroke, and family history of diabetes.

Table 3 shows the prospective association between quintile groups of proportional less-healthy food consumption and mortality. The unadjusted analyses show an association between less-healthy food consumption and cardiovascular mortality. After adjustment for sociodemographic and behavioural factors (Model 1), there was an apparent inverse (protective) association (test for trend, *p* = 0.03) between consumption of less-healthy food and cardiovascular mortality. After additional adjustment for indicators of cardiovascular risk at baseline (Model 2), there was no association between less-healthy food consumption and cardiovascular mortality.

**Table 3:** Hazard ratios for cardiovascular and all-cause mortality by quintile group of proportional less-healthy food consumption in the European Prospective Investigation of Cancer (EPIC)-Norfolk (*n* = 24,880).

		Quintile group of consumption of less-healthy food and beverages					Test for linear trend
		Q1—lowest (*n* = 4,760)	Q2 (*n* = 4,760)	Q3 (*n* = 4,760)	Q4 (*n* = 4,760)	Q5—highest (*n* = 4,759)	
Proportion of energy consumed from foods and beverages categorised as less-healthy (Range, %)		<37.1	37.1–44.4	44.4–50.2	50.2–57.0	57.0–92.7	
Cardiovascular mortality	Deaths	484	497	493	551	530	
	Unadjusted	1.00	1.02 (0.90–1.17)	1.02 (0.90–1.16)	1.07 (0.94–1.21)	1.12 (0.99–1.27)	**<0.001**
	Model 1	1.00	0.91 (0.80–1.04)	**0.84 (0.74–0.96)**	**0.86 (0.76–0.99)**	**0.86 (0.75–0.98)**	0.03
	Model 2	1.00	0.94 (0.82–1.07)	0.92 (0.80–1.04)	0.96 (0.84–1.09)	0.99 (0.87–1.14)	0.90
All-cause mortality	Deaths	1,268	1,379	1,389	1,493	1,610	
	Unadjusted	1.00	**1.09 (1.00–1.18)**	**1.10 (1.01–1.19)**	**1.19 (1.10–1.29)**	**1.31 (1.21–1.41)**	**<0.001**
	Model 1	1.00	0.99 (0.91–1.07)	0.94 (0.87–1.02)	1.00 (0.93–1.10)	1.04 (0.96–1.13)	0.25
	Model 2	1.00	0.99 (0.92–1.08)	0.98 (0.90–1.06)	1.05 (0.97–1.14)	**1.11 (1.02–1.20)**	**0.006**

Model 1 is adjusted for age, sex, alcohol consumption, physical activity, smoking status, education level, and total dietary energy. Model 2 is adjusted for Model 1 covariates plus blood pressure-lowering medication, lipid-lowering medication, past heart attack, past stroke, angina, diabetes, hypertension, hypercholesterolemia, past cancer diagnosis, family history of heart attack, family history of stroke, and family history of diabetes.

The unadjusted analyses showed an association between less-healthy food consumption and all-cause mortality. After adjustment for sociodemographic risk factors and behavioural risk factors (Model 1), there was no association. After further adjustment for indicators of cardiovascular risk at baseline (Model 2), a higher risk of all-cause mortality was observed for those in quintile group 5 relative to those in quintile group 1 (hazard ratio = 1.11, 95% CI 1.02–1.20).

An inverse (protective) association between fruit and vegetable consumption (quintile group of consumption by weight) and incident CVD was observed, in unadjusted and all adjusted models (Table 4).

**Table 4:** Hazard ratios of incident cardiovascular disease by quintile group of proportional fruit and vegetable consumption in the European Prospective Investigation of Cancer (EPIC)-Norfolk (*n* = 22,992).

	Quintile group of fruit and vegetable consumption (proportion of food weight consumed as fruit and vegetables)					
	Q1—lowest (*n* = 4599)	Q2 (*n* = 4598)	Q3 (*n* = 4,599)	Q4 (*n* = 4,598)	Q5—highest (*n* = 4,598)	Test for linear trend
Weight of fruit and vegetables (Range, g/d)	0–185	185–262	262–339	339–448	448–2,441	
Cases	1,092	1,021	1,003	959	890	
Unadjusted model	1.00	**0.91 (0.83–0.99)**	**0.88 (0.81–0.96)**	**0.83 (0.76–0.91)**	**0.76 (0.69–0.83)**	**<0.001**
Model 1	1.00	0.94 (0.86–1.03)	0.95 (0.87–1.03)	0.93 (0.85–1.02)	**0.88 (0.80–0.96)**	**0.01**
Model 2	1.00	0.92 (0.84–1.00)	0.92 (0.84–1.00)	**0.89 (0.81–0.97)**	**0.84 (0.76–0.92)**	**<0.001**

Model 1 is adjusted for age, sex, alcohol consumption, physical activity, smoking status, education level, and total dietary energy. Model 2 is adjusted for Model 1 covariates plus blood pressure-lowering medication, lipid-lowering medication, diabetes, hypertension, hypercholesterolemia, past cancer diagnosis, family history of heart attack, family history of stroke, and family history of diabetes.

### Sensitivity analyses

After adjustment (Model 2), no association was observed for the separate outcomes of incident stroke and incident myocardial infarction (Table C in S1 Data). When not adjusting for total dietary energy intake, an inverse (protective) association between quintile of less-healthy food consumption and risk of incident CVD was observed for Model 1, and no association was observed for Model 2 (Table D in S1 Data)

After adjustment (Model 1 and Model 2), no association was observed between proportional less-healthy food consumption (based on proportion of food weight that was categorised as less-healthy) and incident CVD (Table E in S1 Data), nor was an association observed between less-healthy food consumption (Model 2), based on the mean energy-weighted score of all items consumed, and incident CVD (Table F in S1 Data). The ‘substitution model’ indicates that the isocaloric replacement of less-healthy food for healthier food (or vice versa) was not associated with increased risk of CVD (Model 1 and Model 2, Table G in S1 Data).

Further sensitivity analyses attempted to deal with possible reverse causation. Additional adjustment for BMI did not materially alter the findings (Table B in S1 Data), nor did exclusion of participants with comorbid conditions at baseline (*n* = 21,338 for exclusion of diabetes and cancer, Table H in S1 Data, and *n* = 17,948 for exclusion of participants with self-reported hypertension or hyperlipidaemia or blood pressure- or lipid-lowering medication, Table I in S1 Data), participants with a family history of CVD (*n* = 11,481, Table J in S1 Data), or participants who experienced an incident event within 2 years of follow-up (*n* = 22,737, Table K in S1 Data). The findings were also similar when excluding participants with comorbid conditions or a family history of CVD or who experienced an incident event within 2 years of follow-up (Table L in S1 Data).

Further exclusion of prevalent diseases for the mortality analyses did not appreciably alter the findings (Table M in S1 Data).

## Discussion

In this population-based cohort of older UK adults, we did not detect any significant association between the quantity of less-healthy food consumed, defined using the FSA-Ofcom model, and incident CVD or cardiovascular mortality after adjustment for confounders. Whilst unadjusted models showed positive and significant associations between the quantity of less-healthy food consumed and cardiovascular outcomes (Tables 2 and 3), this was explained by a number of confounding factors, principally age and sex, and as such, we do not consider these crude associations to be meaningful. There was also a suggestion that those who report lower intakes of less-healthy foods were at higher risk of CVD (e.g., high prevalence of diabetes and medication usage among participants in quintile group 1; see Table 1). For this reason, we put more emphasis on the findings of Model 2, which adjusts for indicators of cardiovascular risk at baseline, when considering cardiovascular outcomes.

We did observe an association between less-healthy food consumption and all-cause mortality after adjustment for baseline indicators of cardiovascular risk (Model 2), but not when only adjusting for sociodemographic and behavioural risk factors (Model 1). Given that CVD accounts for a third of all deaths (35.7%) and the absence of associations for CVD, it might be more appropriate to put greater emphasis on the Model 1 findings for the all-cause mortality analyses. Given this and having undertaken multiple tests of significance, we suggest the all-cause mortality Model 2 findings should be treated with caution.

The key strength of this study is defining the exposure in a way that reflects the operational usage of the FSA-Ofcom model in the UK, making use of 7-day diet diaries, which in our sample have been shown to have greater agreement with objective measures of diet than other common methods (24-hour recall or food frequency questionnaires) [19,30]. We have tested associations with both specific outcomes (ischaemic heart disease and stroke), for which there is a greater a priori expectation of an association given the components included in the FSA-Ofcom model, and nonspecific outcomes (all-cause mortality). While these are important health outcomes, we note they are not health outcomes observed in children, who are the intended beneficiaries of the restriction of television advertising of less-healthy food.

Dietary behaviour is self-reported and may be inaccurate or biased. Baseline dietary data were collected in the 1990s. The foods on offer in the 1990s, particularly processed foods, may not reflect the foods that people consume today in the UK or elsewhere. Our study has effectively tested the FSA-Ofcom model across all foods in the diet, whereas the scoring system is only likely to be operationalised (in the UK) on those foods that are heavily advertised (i.e., manufactured or processed foods).

Our findings are notably different to recently published findings from two French cohort studies SU.VI.MAX (SUpplementation en VItamines et MinérauxAntioXydants) (*n* = 13,017) and NutriNet-Santé (*n* = 75,801) [11–14]. Whilst some of the outcomes in these publications (e.g., cancer) are different to our primary outcome, others are related (e.g., metabolic syndrome and weight gain) or the same (incident CVD). Besides some differences in the scoring system for cheese, fats, and beverages [13,15], there are a number of differences between the studies in terms of population (the French cohorts are younger, with a mean age of 48.9 and 43.1 years, respectively, and have experienced relatively fewer events, with 511 incident cases of metabolic syndrome and 509 major CVD events, respectively) and dietary ascertainment (the French cohorts both used repeated 24-hour recall) [10,13,14,31]. Habitual differences in diet may also contribute to differences in the finding [32,33]. It should also be noted that the scoring system is operationalised slightly differently in France, e.g., with adjustments made for diet drinks and soft cheeses.

Apart from the studies based on SU.VI.MAX and NutriNet-Santé, the Whitehall II study tested the association between the FSA-Ofcom nutrient profile model and CVD risk. However, this study focused on dietary variety (rather than quantity of consumption of less-healthy food) and found that total food variety and variety of recommended (‘healthy’) foods (but not nonrecommended foods) were associated with reduced coronary heart disease mortality and cancer morality, respectively.[34]

There are several possible explanations for the absence of an association between the FSA-Ofcom model and prospective CVD in the adjusted analyses in our study. First, our findings could be a ‘false negative’, either because of chance or because of limited power. However, fruit and vegetable consumption (as a proportion of total food energy) was significantly associated with CVD, and other EPIC-Norfolk studies have detected significant associations between dietary indices (e.g., Mediterranean Diet Score) or dietary factors (e.g., fish consumption) and incident CVD [35–37]. This suggests that the study should have sufficient power. Nonetheless, we note that the point estimate and confidence intervals observed are still consistent with a small increased hazard ratio for people in quintile group 5 compared to those in quintile group 1—i.e., the FSA-Ofcom model may be weakly associated with disease.

Second, the failure to find an association may reflect insufficient heterogeneity between the quintile groups, although quintile 5 participants consumed approximately 3 times as much less-healthy food (by weight and energy) as quintile group 1. We also note the variation in mean energy-weighted score of food (3.9 in quintile group 1 to 10.1 in quintile group 5) was greater than that observed in the French cohort, so insufficient heterogeneity seems an unlikely reason for our null findings [13]. Third, reverse causation may be a factor. We note that participants in quintile group 1 (lowest proportion of less-healthy food) appeared to be at higher cardiovascular risk (as indicated by medication, family history, and BMI). This might suggest that participants in quintile group 1 were at higher risk of CVD and were choosing to adopt a healthier eating pattern to offset this risk. Although we undertook extensive analyses to account for reverse causation, both adjustment and exclusion, we cannot rule out residual confounding and reverse causation as an explanation for our findings. As some of the covariates that we adjusted for (e.g., diagnosis of high blood pressure) could be on the causal pathway between less-healthy food consumption and CVD, adjustment for these risk factors might have attenuated a hypothetical association between less-healthy food consumption and increased incidence of CVD. However, we did not observe any associations when we excluded these risk factors from our analyses (i.e., restricted the analysis to participants who did not have indices of increased cardiovascular risk at baseline).

Finally, it is possible that our findings indicate a ‘true negative’, i.e., the FSA-Ofcom model is not, or is only weakly, associated with CVD, reflecting potential shortcomings of the model. The model was published in 2004, prior to some key advances in nutritional science [38]. The notion that saturated fat consumption is a risk factor for CVD has been challenged [39,40]. The FSA-Ofcom model may misclassify some foods because it does not account for the cardioprotective effects of mono- and polyunsaturated fats, classifying all oils, including healthier oils (e.g., olive oil) as less-healthy. The model also fails to discriminate between some healthy and less-healthy grains, e.g., between brown and white rice or between wholemeal and white bread [38]. This may explain why estimated fibre intake was not strongly patterned across the quintiles (see Table 1) despite the inclusion of fibre within the scoring algorithm. We note the FSA-Ofcom model is presently being reviewed in light of revised dietary guidelines on sugar intake [41].

While no single study is definitive, our findings do call into question the FSA-Ofcom model’s value for public health, particularly in the UK. We should emphasise that our analysis amounts primarily to an evaluation of the model’s classification of less-healthy foods, not the underlying scoring system. One should be cautious about extrapolating our findings to other variants of the FSA-Ofcom model (e.g., the New Zealand, Australian, or French versions) that apply different scoring and classification systems. We also want to emphasize that our study was not designed to test current dietary guidelines or advice around the consumption of specific ‘unhealthy’ foods. There is a robust evidence base concerning the health risks associated with the consumption of many such food groups (e.g., red meat, sugar-sweetened beverages, and takeaway food) [42–46]. On the basis of our study, it would be inappropriate to conclude that the present dietary advice about the consumption of certain foods, some of which may be labelled unhealthy or less-healthy, is incorrect.

Given the conflicting findings of our study and those based on a French cohort [11–14], further replications in other cohorts and considering other outcomes (e.g., weight change) would be of value. However, the failure to demonstrate a positive association between less-healthy food consumption and CVD in this cohort suggests the FSA-Ofcom model is not consistently discriminating among foods with respect to their association with CVD in the UK context. It may be appropriate for public health officials and scientists to review whether and how the FSA-Ofcom model could be improved for use in the UK and elsewhere, but it would not be appropriate to use the study to undermine present dietary advice.

## Supporting information

S1 Data Supplementary data. (DOCX)

S1 STROBE Checklist Strengthening the reporting of observational studies in epidemiology (STROBE) statement. (DOC)

S1 Text Study proposal. (DOCX)

## Abbreviations

BMI: body mass index
CVD: cardiovascular disease
EPIC: European Prospective Investigation of Cancer
FSA: Food Standards Agency
SU.VI.MAX: SUpplementation en VItamines et MinérauxAntioXydants
