# Supplementary material for: Association between intake of less-healthy foods defined by the United Kingdom's nutrient profile model and cardiovascular disease: A population-based cohort study
Source: PLoS Med. 2018 Jan 4;15(1):e1002484. doi: 10.1371/journal.pmed.1002484 (PMC5754044; doi:10.1371/journal.pmed.1002484)
Supplement: S1 Data — (DOCX) [file pmed.1002484.s001.docx]

**Table A: Descriptive characteristics of participants included in the analysis and those excluded due to missing co-variates**

|  | **Included**  **(n=22992)** | | | **Excluded**  **(n=250)** | | |  |
| --- | --- | --- | --- | --- | --- | --- | --- |
| Age (years) | 58.6 (9.2) | | | 62.4 (9.0) [n=250] | | |  |
| Women (%) | 56.5 | | | 60.0 [n=250] | | |  |
| Education: Degree or higher (%) | 13.4 | | | 10.0 [n=250] | | |  |
| Current smoker (%) | 11.8 | | | 12.7 [n=71] | | |  |
| Physical activity: active (%) | 18.7 | | | 14.8 [n=250] | | |  |
| Past cancer diagnosis (%) | 5.4 | | | 6.1 [n=246] | | |  |
| Diabetes (%) | 1.9 | | | 2.1 [n=242] | | |  |
| Family history of heart attack (%) | 35.6 | | | 31.4 [n=245] | | |  |
| Anti-hypertensive medication (%) | 14.9 | | | 22.4 [n=250] | | |  |
| Lipid lowering medication (%) | 0.9 | | | 0.0 [n=250] | | |  |
| BMI (kg/m^2^) | 26.3 (3.9) | | | 26.8 (4.2) [n=250] | | |  |
| Systolic blood pressure (mmHg) | 135.2 (18.4) | | | 140.0 (19.4) [n=250] | | |  |
| Total cholesterol (mmol/L) | 6.17 (1.16) | | | 6.25 (1.20) [n=226) | | |  |
| HDL cholesterol (mmol/L) | 1.43 (0.42) | | | 1.40 (0.40) [n=220] | | |  |
| Fruit (g) | 173 (134) | | | 166 (122) [n=250] | | |  |
| Vegetables (g) | 152 (77) | | | 140 (77) [n=250] | | |  |
| Fish (g) | 27.4 (27.4) | | | 26.1 (24.0) [n=250] | | |  |
| Processed meat (g) | 22.3 (21.0) | | | 23.9 (30.2) [n=250] | | |  |
| Alcohol (units) | 1.51 (2.21) | | | 1.16 (2.10) [n=250] | | |  |
| Energy (kJ) | 8217 (2192) | | | 7978 (2339) [n=250] | | |  |
| Percentage of energy from saturated fat (%) | 12.9 (3.1) | | | 13.0 (3.0) [n=250] | | |  |
| Ratio of saturated unsaturated fat | 2.13 (0.84) | | | 2.28 (1.18) [n=250] | | |  |
| Sodium (mg) | 2760 (850) | | | 2680 (802) [n=250] | | |  |
| Fibre (g) | 15.1 (5.5) | | | 14.5 (5.3) [n=250] | | |  |
| Mean energy-weighted nutrient profile score | 6.91 (2.28) | | | 7.27 (2.13) [n=250] | | |  |
| Less-healthy food (g/d) | 297.3 (122) | | | 293.4 (112.7) [n=250] | | |  |
| Less-healthy food (kJ/d) | 3718 (1535) | | | 3739 (1533) [n=250] | | |  |
| Healthy food (kJ/d) | 3480 (1010) | | | 3313 (947) [n=250] | | |  |
| Less-healthy beverage (kJ/d) | 216 (342) | | | 264 (419) [n=250] | | |  |
|  | |  |  | |  |  | |
|  | |  |  | |  |  | |
|  | |  |  | |  |  | |
|  | |  |  | |  |  | |
|  | |  |  | |  |  | |
|  | |  |  | |  |  | |
|  | |  |  | |  |  | |
|  | |  |  | |  |  | |
|  | |  |  | |  |  | |
|  | |  |  | |  |  | |
|  | |  |  | |  |  | |
|  | |  |  | |  |  | |
|  | |  |  | |  |  | |
|  | |  |  | |  |  | |
|  | |  |  | |  |  | |
|  | |  |  | |  |  | |
|  | |  |  | |  |  | |
|  | |  |  | |  |  | |

**Table B: Hazard ratios of incident cardiovascular disease by quintile group of proportional less-healthy food consumption in EPIC-Norfolk alternative models (n=22,992)**

|  | **Quintile of ‘less healthy’ food and beverage consumption**  **(proportion of energy consumed from foods and beverages categorised as ‘less healthy’)** | | | | | |
| --- | --- | --- | --- | --- | --- | --- |
|  | **Q1 - lowest consumption**  **(n=4599)** | **Q2**  **(n=4598)** | **Q3**  **(n=4599)** | **Q4**  **(n=4598)** | **Q5 - highest consumption (=4598)** | **Test for trend** |
| Proportion of energy consumed from foods and beverages categorised as less-healthy (Range, %) | 0-37.2 | 37.2-44.5 | 44.5-50.3 | 50.3-57.0 | 57.0-92.7 |  |
| Cases | 706 | 725 | 726 | 719 | 803 | 3,679 |
| Unadjusted model | 1.00 | 1.04 (0.95-1.14) | 1.07 (0.98-1.17) | 1.09 (0.99-1.19) | **1.19 (1.10-1.31)** | **0.007** |
| Model 1 | 1.00 | 0.97 (0.88-1.06) | 0.93 (0.85-1.02) | **0.90 (0.82-0.99)** | 0.93 (0.84-1.03) | **0.009** |
| Model 2 | 1.00 | 0.99 (0.91-1.08) | 0.99 (0.90-1.08) | 0.97 (0.88-1.07) | 1.01 (0.92-1.12) | 0.84 |
| Model 2 + BMI | 1.00 | 0.98 (0.90-1.08) | 0.99 (0.90-1.09) | 0.96 (0.87-1.06) | 1.02 (0.92-1.12) | 0.89 |
| Model 2’ | 1.00 | 0.97 (0.89-1.06) | 0.95 (0.87-1.04) | 0.92 (0.84-1.01) | 0.96 0.87-1.07) | 0.34 |

IHD = ischaemic heart diseases; Model 1 is adjusted for age, sex, alcohol consumption, physical activity, smoking status, education level and total dietary energy; Model 2 is adjusted for Model 1 co-variates plus blood pressure lowering medication, lipid lowering medication, diabetes, hypertension, hypercholesterolemia, past cancer diagnosis, family history of heart attack, family history of stroke and family history of diabetes; Model 2’ is adjusted for Model 1 co-variates plus diabetes, past cancer diagnosis, family history of heart attack, family history of stroke and family history of diabetes

**Table C: Hazard ratios of incident ischaemic heart disease and stroke by quintile of consumption of foods and beverages categorised as ‘less-healthy’ in EPIC-Norfolk (n=22,992)**

|  |  | **Quintile of ‘less healthy’ food and beverage consumption**  **(proportion of energy consumed from foods and beverages categorised as ‘less healthy’)** | | | | | |
| --- | --- | --- | --- | --- | --- | --- | --- |
|  |  | **Q1 - lowest consumption**  **(n=4599)** | **Q2**  **(n=4598)** | **Q3**  **(n=4599)** | **Q4**  **(n=4598)** | **Q5 - highest consumption (=4598)** | **Test for trend** |
|  | Proportion of energy consumed from foods and beverages categorised as less-healthy (Range, %) | 0-37.2 | 37.2-44.5 | 44.5-50.3 | 50.3-57.0 | 57.0-92.7 |  |
| IHD | Cases | 706 | 725 | 726 | 719 | 803 | 3,679 |
|  | Unadjusted model | 1.00 | 1.03 (0.93-1.14) | 1.04 (0.93-1.15) | 1.03 (0.93-1.14) | **1.18 (1.06-1.3)** | **0.004** |
|  | Model 1 | 1.00 | 0.95 (0.86-1.06) | 0.90 (0.81-1.01) | **0.85 (0.76-0.95)** | 0.91 (0.81-1.02) | **0.03** |
|  | Model 2 | 1.00 | 0.98 (0.88-1.09) | 0.97 (0.87-1.08) | 0.93 (0.83-1.03) | 1.00 (0.89-1.12) | 0.69 |
| Stroke | Cases | 330 | 355 | 377 | 398 | 409 | 1869 |
|  | Unadjusted model | 1.00 | 1.08 (0.93-1.25) | 1.15 (0.99-1.33) | **1.23 (1.06-1.43)** | **1.29 (1.11-1.49)** | **<0.001** |
|  | Model 1 | 1.00 | 0.97 (0.83-1.12) | 0.96 (0.82-1.12) | 0.98 (0.84-1.15) | 0.98 (0.83-1.15) | 0.93 |
|  | Model 2 | 1.00 | 0.98 (0.84-1.14) | 1.00 (0.86-1.17) | 1.04 (0.89-1.22) | 1.03 (0.88-1.22) | 0.46 |

IHD = ischaemic heart diseases; Model 1 is adjusted for age, sex, alcohol consumption, physical activity, smoking status, education level and total dietary energy; Model 2 is adjusted for Model 1 co-variates plus blood pressure lowering medication, lipid lowering medication, diabetes, hypertension, hypercholesterolemia, past cancer diagnosis, family history of heart attack, family history of stroke and family history of diabetes.

**Table D: Hazard ratios of incident cardiovascular disease by quintile of consumption of foods and beverages categorised as ‘less-healthy’ excluding participants without adjustment for total dietary energy (n=22992)**

|  | **Quintile of ‘less healthy’ food and beverage consumption**  **(proportion of energy consumed from foods and beverages categorised as ‘less healthy’)** | | | | | |
| --- | --- | --- | --- | --- | --- | --- |
|  | **Q1 - lowest**  **(n=4599)** | **Q2**  **(n=4598)** | **Q3**  **(n=4599)** | **Q4**  **(n=4598)** | **Q5 - highest (n=4598)** | **Test for trend** |
| Proportion of energy consumed from foods and beverages categorised as less-healthy (Range, %) | 0-37.2 | 37.2-44.5 | 44.5-50.3 | 50.3-57.0 | 57.0-92.7 |  |
| Cases | 934 | 971 | 988 | 999 | 1073 | 4965 |
| Unadjusted model | 1.00 | 1.04 (0.95-1.13) | 1.06 (0.97-1.16) | 1.08 (0.98-1.18) | 1.19 (1.09-1.29) | **<0.01** |
| Model 1 | 1.00 | 0.94 (0.86-1.03) | 0.90 (0.82-0.99) | 0.86 (0.78-0.94) | 0.88 (0.8-0.96) | **0.001** |
| Model 2 | 1.00 | 0.96 (0.88-1.05) | 0.96 (0.87-1.05) | 0.92 (0.84-1.01) | 0.95 (0.87-1.05) | 0.20 |

Model 1 is adjusted for age, sex, alcohol consumption, physical activity, smoking status, education level; Model 2 is adjusted for Model 1 co-variates plus blood pressure lowering medication, lipid lowering medication, diabetes, hypertension, hypercholesterolemia, past cancer diagnosis, family history of heart attack, family history of stroke and family history of diabetes.

**Table E: Hazard ratios of incident cardiovascular disease by quintile of consumption of foods weight categorised as ‘less-healthy’ in EPIC-Norfolk (n=22,992)**

|  | **Quintile of ‘less healthy’ food consumption**  **(proportion of food weight consumed from foods categorised as ‘less healthy’)** | | | | | |
| --- | --- | --- | --- | --- | --- | --- |
|  | **Q1 - lowest consumption**  **(n=4599)** | **Q2**  **(n=4598)** | **Q3**  **(n=4599)** | **Q4**  **(n=4598)** | **Q5 - highest consumption (=4598)** | **Test for trend** |
| Proportion of food weight categorised as less-healthy (Range, %) | 0-20.1 | 20.1-25.7 | 24.7-30.7 | 30.7-36.8 | 36.8-93.4 |  |
| Cases | 927 | 978 | 980 | 1020 | 1060 | 4965 |
| Unadjusted model | 1.00 | 1.07 (0.98-1.17) | 1.08 (0.99-1.18) | **1.12 (1.02-1.22)** | **1.20 (1.10-1.31)** | **<0.001** |
| Model 1 | 1.00 | 1.01 (0.92-1.10) | 0.98 (0.89-1.07) | 0.98 (0.89-1.08) | 1.01 (0.92-1.11) | 0.99 |
| Model 2 | 1.00 | 1.03 (0.94-1.13) | 1.01 (0.92-1.11) | 1.04 (0.94-1.14) | 1.07 (0.97-1.17) | 0.22 |

Model 1 is adjusted for age, sex, alcohol consumption, physical activity, smoking status, education level and total dietary energy; Model 2 is adjusted for Model 1 co-variates plus blood pressure lowering medication, lipid lowering medication, past heart attack, past stroke, angina, diabetes, hypertension, hypercholesterolemia, past cancer diagnosis, family history of heart attack, family history of stroke and family history of diabetes.

**Table F: Hazard ratios of incident cardiovascular disease by quintile of dietary quality (mean-energy weighted FSA-Ofcom score) in EPIC-Norfolk (n=22,992)**

|  | **Quintile of ‘dietary quality’**  **(mean-energy weighted FSA-Ofcom score)** | | | | | |
| --- | --- | --- | --- | --- | --- | --- |
|  | **Q1 - lowest consumption**  **(n=4599)** | **Q2**  **(n=4598)** | **Q3**  **(n=4599)** | **Q4**  **(n=4598)** | **Q5 - highest consumption (=4598)** | **Test for trend** |
| Energy weighted FSA-Ofcom score (Range) | -3.15 to 5.04 | 5.04 to 6.43 | 6.43 to 7.53 | 7.53 to 8.80 | 8.81 to 17.15 |  |
| Cases | 928 | 968 | 982 | 1033 | 1054 | 4965 |
| Unadjusted model | 1.00 | 1.06 (0.98-1.17) | 1.08 (0.99-1.18) | **1.16 (1.05-1.26)** | **1.18 (1.08-1.29)** | **<0.001** |
| Model 1 | 1.00 | 0.94 (0.86-1.03) | **0.91 (0.83-1.00)** | 0.94 (0.86-1.04) | **0.90 (0.82-1.00)** | 0.10 |
| Model 2 | 1.00 | 0.98 (0.89-1.07) | 0.96 (0.88-1.06) | 1.02 (0.92-1.12) | 0.98 (0.89-1.08) | 0.96 |

Model 1 is adjusted for age, sex, alcohol consumption, physical activity, smoking status, education level and total dietary energy; Model 2 is adjusted for Model 1 co-variates plus blood pressure lowering medication, lipid lowering medication, past heart attack, past stroke, angina, diabetes, hypertension, hypercholesterolemia, past cancer diagnosis, family history of heart attack, family history of stroke and family history of diabetes.

**Table G: Hazard ratios of incident cardiovascular disease when substituting ‘healthy’ food for other types of dietary energy, holding total energy intake constant in EPIC-Norfolk (n=22,992)**

|  |  | |  |  |
| --- | --- | --- | --- | --- |
| **Category of food or drink** |  | **Hazard Ratio (95% CI)** | |  |
|  | **Unadjusted** | **Model 1** | | **Model 2** |
| Less-healthy food (per 500kJ/day) | **1.03 (1.01-1.05)** | 0.98 (0.96-1.00) | | 0.99 (0.94-1.05) |
| Less-healthy beverages (per 500kJ/day) | **1.05 (1.00-1.10)** | 0.98 (0.94-1.03) | | 1.01 (0.96-1.05) |
| Healthy beverages (per 500kJ/day) | 0.96 (0.91-1.01) | 1.01 (0.95-1.06) | | 0.99 (0.94-1.05) |
| Alcohol (per 500kJ/day) | 0.99 (0.97-1.03) | 0.99 (0.83-1.19) | | 1.01 (0.84-1.20) |
| Total dietary energy (per 500kJ/day) | **0.97 (0.96-0.99)** | 1.00 (0.98-1.01) | | 0.99 (0.98-1.01 |

‘Healthy’ food are items of food or drink that are not categorised as ‘less-healthy’ food. The reported hazard ratios for the unadjusted model are mutually adjusted for all co-variates listed in the table, i.e. there is no adjustment for the covariates included in Model 1 and Model 2; Model 1 is adjusted for age, sex, alcohol consumption, physical activity, smoking status, education level and total dietary energy; Model 2 is adjusted for Model 1 co-variates plus blood pressure lowering medication, lipid lowering medication, past heart attack, past stroke, angina, diabetes, hypertension, hypercholesterolemia, past cancer diagnosis, family history of heart attack, family history of stroke and family history of diabetes.

**Table H: Hazard ratios of incident cardiovascular disease by quintile of consumption of foods and beverages categorised as ‘less-healthy’ excluding participants with self-reported diabetes or past cancer diagnosis in EPIC-Norfolk (n=21,338)**

|  | **Quintile of ‘less healthy’ food and beverage consumption**  **(proportion of energy consumed from foods and beverages categorised as ‘less healthy’)** | | | | | |
| --- | --- | --- | --- | --- | --- | --- |
|  | **Q1 - lowest consumption**  **(n=4210)** | **Q2**  **(n=4252)** | **Q3**  **(n=4281)** | **Q4**  **(n=4290)** | **Q5 - highest consumption (=4305)** | **Test for trend** |
| Proportion of energy consumed from foods and beverages categorised as less-healthy (Range, %) | 0-37.2 | 37.2-44.5 | 44.5-50.3 | 50.3-57.0 | 57.0-92.7 |  |
| Cases | 801 | 866 | 903 | 909 | 986 | 4465 |
| Unadjusted model | 1.00 | 1.07 (0.98-1.18) | 1.12 (1.02-1.23) | 1.12 (1.02-1.23) | 1.25 (1.14-1.37) | **<0.001** |
| Model 1 | 1.00 | 0.98 (0.89-1.08) | 0.96 (0.87-1.06) | 0.91 (0.83-1.01) | 0.96 (0.86-1.06) | 0.23 |
| Model 2 | 1.00 | 0.99 (0.90-1.09) | 1.00 (0.91-1.10) | 0.94 (0.85-1.05) | 1.00 (0.90-1.12) | 0.78 |

Model 1 is adjusted for age, sex, alcohol consumption, physical activity, smoking status, education level and total dietary energy; Model 2 is adjusted for Model 1 co-variates plus blood pressure lowering medication, lipid lowering medication, hypertension, hypercholesterolemia, family history of heart attack, family history of stroke and family history of diabetes.

**Table I: Hazard ratios of incident cardiovascular disease by quintile of consumption of foods and beverages categorised as ‘less-healthy’ excluding participants who reported a diagnosis of hypertension or hyperlipidemia or who reported use of blood pressure or lipid lowering medication in EPIC-Norfolk (n=17948)**

|  | **Quintile of ‘less healthy’ food and beverage consumption**  **(proportion of energy consumed from foods and beverages categorised as ‘less healthy’)** | | | | | |
| --- | --- | --- | --- | --- | --- | --- |
|  | **Q1 - lowest consumption**  **(n=3438)** | **Q2**  **(n=3499)** | **Q3**  **(n=3625)** | **Q4**  **(n=3664)** | **Q5 - highest consumption (=3722)** | **Test for trend** |
| Proportion of energy consumed from foods and beverages categorised as less-healthy (Range, %) | 0-37.2 | 37.2-44.5 | 44.5-50.3 | 50.3-57.0 | 57.0-92.7 |  |
| Cases | 562 | 608 | 666 | 681 | 756 | 3273 |
| Unadjusted model | 1.00 | 1.06 (0.95-1.18) | 1.13 (1.02-1.25) | 1.15 (1.03-1.29) | 1.28 (1.15-1.43) | **<0.001** |
| Model 1 | 1.00 | 0.96 (0.86-1.08) | 0.94 (0.86-1.07) | 0.90 (0.80-1.02) | 0.94 (0.83-1.06) | 0.22 |
| Model 2 | 1.00 | 0.98 (0.88-1.11) | 0.96 (0.86-1.09) | 0.93 (0.83-1.05) | 0.97 (0.86-1.11) | 0.55 |

Model 1 is adjusted for age, sex, alcohol consumption, physical activity, smoking status, education level and total dietary energy; Model 2 is adjusted for Model 1 co-variates plus diabetes, past cancer diagnosis, family history of heart attack, family history of stroke and family history of diabetes.

**Table J: Hazard ratios of incident cardiovascular disease by quintile of consumption of foods and beverages categorised as ‘less-healthy’ excluding participants who reported a family history of stroke of heart attack (n=11481)**

|  | **Quintile of ‘less healthy’ food and beverage consumption**  **(proportion of energy consumed from foods and beverages categorised as ‘less healthy’)** | | | | | |
| --- | --- | --- | --- | --- | --- | --- |
|  | **Q1 - lowest consumption**  **(n=3438)** | **Q2**  **(n=3499)** | **Q3**  **(n=3625)** | **Q4**  **(n=3664)** | **Q5 - highest consumption (=3722)** | **Test for trend** |
| Proportion of energy consumed from foods and beverages categorised as less-healthy (Range, %) | 0-37.2 | 37.2-44.5 | 44.5-50.3 | 50.3-57.0 | 57.0-92.7 |  |
| Cases | 398 | 418 | 439 | 423 | 524 | 2202 |
| Unadjusted model | 1.00 | 1.03 (0.90-1.18) | 1.08 (0.95-1.24) | 1.07 (0.93-1.22) | 1.31 (1.15-1.49) | **<0.001** |
| Model 1 | 1.00 | 0.93 (0.80-1.07) | 0.89 (0.78-1.02) | 0.83 (0.72-0.96) | 0.95 (0.82-1.10) | 0.31 |
| Model 2 | 1.00 | 0.96 (0.84-1.10) | 0.94 (0.82-1.09) | 0.87 (0.83-1.01) | 1.03 (0.88-1.19) | 0.92 |

Model 1 is adjusted for age, sex, alcohol consumption, physical activity, smoking status, education level and total dietary energy; Model 2 is adjusted for Model 1 co-variates plus blood pressure lowering medication, lipid lowering medication, diabetes, hypertension, hypercholesterolemia, past cancer diagnosis and family history of diabetes

**Table K: Hazard ratios of incident cardiovascular disease by quintile of consumption of foods and beverages categorised as ‘less-healthy’ excluding incident events within two years of follow-up (n=22,737)**

|  | **Quintile of ‘less healthy’ food and beverage consumption**  **(proportion of energy consumed from foods and beverages categorised as ‘less healthy’)** | | | | | |
| --- | --- | --- | --- | --- | --- | --- |
|  | **Q1 - lowest**  **(n=4552)** | **Q2**  **(n=4547)** | **Q3**  **(n=4551)** | **Q4**  **(n=4545)** | **Q5 - highest (n=4542)** | **Test for trend** |
| Proportion of energy consumed from foods and beverages categorised as less-healthy (Range, %) | 0-37.2 | 37.2-44.5 | 44.5-50.3 | 50.3-57.0 | 57.0-92.7 |  |
| Cases | 914 | 950 | 968 | 975 | 1,045 | 4852 |
| Unadjusted model | 1.00 | 1.04 (0.95-1.14) | 1.06 (0.97-1.17) | 1.07 (0.98-1.18) | 1.18 (1.08-1.29) | **<0.001** |
| Model 1 | 1.00 | 0.95 (0.87-1.05) | 0.92 (0.84-1.01) | 0.88 (0.8-0.97) | 0.92 (0.83-1.01) | **0.04** |
| Model 2 | 1.00 | 0.97 (0.89-1.07) | 0.98 (0.89-1.08) | 0.95 (0.86-1.04) | 0.99 (0.9-1.1) | 0.72 |

Model 1 is adjusted for age, sex, alcohol consumption, physical activity, smoking status, education level; Model 2 is adjusted for Model 1 co-variates plus blood pressure lowering medication, lipid lowering medication, diabetes, hypertension, hypercholesterolemia, past cancer diagnosis, family history of heart attack, family history of stroke and family history of diabetes.

**Table L: Hazard ratios of incident cardiovascular disease by quintile of consumption of foods and beverages categorised as ‘less-healthy’ excluding participants with incident events within two years of follow-up, co-morbid conditions at baseline or a family history of cardio-metabolic disease (n=7,841)**

|  | **Quintile of ‘less healthy’ food and beverage consumption**  **(proportion of energy consumed from foods and beverages categorised as ‘less healthy’)** | | | | | |
| --- | --- | --- | --- | --- | --- | --- |
|  | **Q1 - lowest**  **(n=1482)** | **Q2**  **(n=1569)** | **Q3**  **(n=4551)** | **Q4**  **(n=1566)** | **Q5 - highest (n=1653)** | **Test for trend** |
| Proportion of energy consumed from foods and beverages categorised as less-healthy (Range, %) | 0-37.2 | 37.2-44.5 | 44.5-50.3 | 50.3-57.0 | 57.0-92.7 |  |
| Cases | 201 | 234 | 251 | 243 | 319 | 1248 |
| Unadjusted model | 1.00 | 1.12 (0.93-1.35) | 1.21 (1.01-1.46) | 1.16 (0.96-1.40) | 1.49 (1.25-1.78) | **<0.001** |
| Model 1 | 1.00 | 0.97 (0.80-1.18) | 0.97 (0.80-1.18) | 0.88 (0.72-1.07) | 1.06 (0.87-1.30) | 0.80 |

Model 1 is adjusted for age, sex, alcohol consumption, physical activity, smoking status, education level; co-morbid conditions excluded are diabetes, cancer, hypertension and hyperlipdemia as well as reported use of blood pressure lowering medication or lipid lowering medication; family history of cardio-metabolic disease includes a family history of diabetes, heart attack or stroke.

**Table M: Hazard ratios for cardiovascular mortality and all-cause mortality by quintile of proportional less-healthy food consumption restricted to participants without previous cardiovascular disease in EPIC-Norfolk (n=22,992)**

|  |  | **Quintile of consumption of less-healthy food and beverages** | | | | | **Test for trend** |
| --- | --- | --- | --- | --- | --- | --- | --- |
|  |  | **Q1 - lowest**  **(n=4599)** | **Q2**  **(n=4598)** | **Q3**  **(n=4599)** | **Q4**  **(n=4598)** | **Q5 - highest (n=4598)** |  |
| Proportion of energy consumed from foods and beverages categorised as less-healthy (Range, %) |  | <37.1 | 37.1-44.4 | 44.4-50.2 | 50.2-57.0 | 57.0-92.7 |  |
| Cardiovascular mortality | Deaths | 336 | 331 | 347 | 383 | 413 |  |
|  | Unadjusted | 1.00 | 0.99 (0.85-1.15) | 1.04 (0.89-1.20) | 1.14 (0.98-1.32) | 1.24 (1.07-1.43) | **<0.001** |
|  | Model 1 | 1.00 | 0.88 (0.76-1.03) | 0.86 (0.73-1.00) | 0.90 (0.77-1.05) | 0.92 (0.78-1.08) | 0.52 |
|  | Model 2 | 1.00 | 0.91 (0.78-1.06) | 0.95 (0.81-1.11) | 1.00 (0.85-1.17) | 1.03 (0.87-1.21) | 0.38 |
| All-cause mortality | Deaths | 1,038 | 1,120 | 1,152 | 1,273 | 1,396 |  |
|  | Unadjusted | 1.00 | 1.08 (0.99-1.18) | 1.11 (1.02-1.21) | 1.23 (1.13-1.33) | 1.35 (1.25-1.47) | **<0.001** |
|  | Model 1 | 1.00 | 0.99 (0.91-1.08) | 0.96 (0.88-1.04) | 1.02 (0.93-1.11) | 1.07 (0.98-1.18) | 0.07 |
|  | Model 2 | 1.00 | 1.00 (0.92-1.09) | 0.99 (0.91-1.08) | 1.06 (0.97-1.16) | 1.12 (1.03-1.23) | **0.004** |

Model 1 is adjusted for age, sex, alcohol consumption, physical activity, smoking status, education level and total dietary energy; Model 2 is adjusted for Model 1 co-variates plus blood pressure lowering medication, lipid lowering medication, past heart attack, past stroke, angina, diabetes, hypertension, hypercholesterolemia, past cancer diagnosis, family history of heart attack, family history of stroke and family history of diabetes.
