# Supplementary material for: Association between intake of less-healthy foods defined by the United Kingdom's nutrient profile model and cardiovascular disease: A population-based cohort study
Source: PLoS Med. 2018 Jan 4;15(1):e1002484. doi: 10.1371/journal.pmed.1002484 (PMC5754044; doi:10.1371/journal.pmed.1002484)
Supplement: S1 Text — (DOCX) [file pmed.1002484.s003.docx]

**Research Proposal: Testing the validity of a nutrient profiling model using EPIC-Norfolk**

**Lead investigator:** Pablo Monsivais (pm491@medschl.cam.ac.uk)

**Co-investigators:** Oliver Mytton (oliver.mytton@dph.ox.ac.uk), Nita Forouhi, Peter Scarborough (University of Oxford) and Mike Rayner (University of Oxford)

**Aim:** To test the validity of the UK (FSA/OfCom) Nutrient Profiling Model by examining its association with incident cardiovascular disease

Exposure measures

The WXYfm model awards points to each food item based on seven characteristics: energy; saturated fat; sugar; fibre; sodium; fruit, vegetables & nuts. The principal food items (defined as “meals” from the principle FFQ used in EPIC) would each be scored according to nutrient content using the WXYfm model.

Each meal can only take an integer value, in the range – 12 to +29. For each individual the proportion of food (by mass; and by energy) for each nutrient profile score will be estimated.

Although the same scoring system is used for drinks and food, the recommended cut-offs are different for food and drink, reflecting the much lower calorie density of drinks. Consequently this process will be done separately for drinks and food. Two sets of scores will be created one for food and one for drinks. Drinks with alcohol will be excluded, as the system was not designed to classify these drinks.

Food will then be categorised into ‘more healthy’ and ‘less healthy food based on the cut-offs used by FSA-Ofcom. Participants will be ordered from low to high based on the proportion of less healthy food consumed in that category. Participants will then be divided into groups (e.g. qunitiles) based on consumption.

Two weighting measures will be used, one by energy (primary measure) and one by energy (secondary measure).

Outcome Measures

The following health outcomes will be examined (incident events):

- Cardiovascular disease (CHD and stroke)
- Cardiovascular mortality
- Total Mortality

Analytic Strategy

1. Descriptive analysis outlining the baseline characteristics (including measures of diet quality) by quintile of exposure to each food category
2. Descriptive analysis outlining the baseline characteristics by quintile of exposure to each food category
3. To adjust for socio-demographic factors, other behaviours and cardiovascular risk, guided by descriptive analyses.
